# Supplementary material for: Depression and anxiety symptoms in young adults before and during the COVID-19 pandemic: evidence from a Canadian population-based cohort
Source: Ann Gen Psychiatry. 2021 Sep 8;20:42. doi: 10.1186/s12991-021-00362-2 (PMC8424412; doi:10.1186/s12991-021-00362-2)
Supplement: Supplementary file 1 — Additional file 1: Table S1. Change in depression and anxiety symptoms from before to during the COVID-19 pandemic. Table S2. Change in depression and anxiety symptoms from before the pandemic to during the pandemic according to different levels of symptoms severity before the pandemic. [file 12991_2021_362_MOESM1_ESM.docx]

**Supplemental Table 1.** Change in depression and anxiety symptoms from before to during the COVID-19 pandemic.

| **Variables** | **Mean difference: depressive symptoms (SE)** | ***p* value** | **Mean difference: anxiety symptoms (SE)** | ***p* value** |
| --- | --- | --- | --- | --- |
| **COVID-19 questionnaire** |  |  |  |  |
| Living alone | 0.22 (0.10) | .020 | 0.05 (0.12) | .657 |
| Loss of employment | 0.06 (0.07) | .451 | 0.12 (0.07) | .107 |
| Loss of education | 0.02 (0.08) | .769 | 0.00 (0.08) | .959 |
| Positive COVID-19 test | 0.10 (0.37) | .795 | 0.03 (0.24) | .917 |
| Frequent Daily COVID-related news seeking | 0.02 (0.07) | .772 | -0.04 (0.07) | .560 |
| Living in Montreal | 0.16 (0.09) | .063 | 0.10 (0.09) | .262 |
| **Pre-existing vulnerabilities** |  |  |  |  |
| Not in education or employed | -0.22 (0.12) | .060 | -0.09 (0.11) | .398 |
| Low family SES | 0.04 (0.09) | .686 | 0.10 (0.09) | .239 |
| Sexual orientation minority | 0.05 (0.13) | .680 | 0.10 (0.13) | .435 |
| Learning disability diagnosis | -0.03 (0.14) | .824 | -0.04 (0.13) | .770 |
| Low social support | -0.01 (0.10) | .924 | 0.04 (0.10) | .712 |
| Low life satisfaction | -0.09 (0.11) | .428 | -0.18 (0.12) | .118 |
| Severe pre-existing depression | -1.26(0.15) | <.001 | -0.62 (0.16) | <.001 |
| Severe pre-existing anxiety | -0.48 (0.20) | .016 | -1.61 (0.15) | <.001 |

*Note*: Data were compiled from the final master file of the Québec Longitudinal Study of Child Development (1998–2020), Québec Government, Québec Statistic Institute. Positive scores indicate a deterioration of mental health.

**Supplemental Table 2.** Change in depression and anxiety symptoms from before the pandemic to during the pandemic according to different levels of symptoms severity before the pandemic.

| **Symptom Severity Before the Pandemic** | **Mean difference: depressive symptoms (SE)** | ***p* value** | **Mean difference: anxiety symptoms (SE)** | ***p* value** |
| --- | --- | --- | --- | --- |
| Very Low (0-20%) | 0.49 (0.05) | <.001 | 0.38 (0.04) | <.001 |
| Low (21-40%) | 0.32 (0.06) | <.001 | 0.28 (0.06) | <.001 |
| Average (41-60%) | 0.07 (0.07) | .322 | 0.02 (0.05) | .654 |
| High (61-80%) | -0.14 (0.07) | .046 | -0.25 (0.08) | .002 |
| Very High (81-100%) | -0.68 (0.08) | <.001 | -0.96 (0.08) | <.001 |

*Note*: Data were compiled from the final master file of the Québec Longitudinal Study of Child Development (1998–2020), Québec Government, Québec Statistic Institute. Positive scores indicate a deterioration of mental health.
